# Connective Tissue Reflex Massage for Type 2 Diabetic Patients with Peripheral Arterial Disease: Randomized Controlled Trial

**DOI:** 10.1093/ecam/nep171

**Published:** 2011-03-13

**Authors:** Adelaida María Castro-Sánchez, Carmen Moreno-Lorenzo, Guillermo A. Matarán-Peñarrocha, Belen Feriche-Fernández-Castanys, Genoveva Granados-Gámez, José Manuel Quesada-Rubio

**Affiliations:** ^1^Department of Nursing and Physical Therapy, University of Almeria, 04120 Almería, Spain; ^2^Department of Physiotherapy, University of Granada, Spain; ^3^Health District of La Vega, Andalusian Health Service (Málaga), Spain; ^4^Department of Physical Education, University of Granada, Spain; ^5^Department of Nursing and Physical Therapy, University of Almeria, Spain; ^6^Department of Statistics, University of Granada, Spain

## Abstract

The objective of this study was to evaluate the efficacy of connective tissue massage to improve blood circulation and intermittent claudication symptoms in type 2 diabetic patients. A randomized, placebo-controlled trial was undertaken. Ninety-eight type 2 diabetes patients with stage I or II-a peripheral arterial disease (PAD) (Leriche-Fontaine classification) were randomly assigned to a massage group or to a placebo group treated using disconnected magnetotherapy equipment. Peripheral arterial circulation was determined by measuring differential segmental arterial pressure, heart rate, skin temperature, oxygen saturation and skin blood flow. Measurements were taken before and at 30 min, 6 months and 1 year after the 15-week treatment. After the 15-week program, the groups differed (*P* < .05) in differential segmental arterial pressure in right lower limb (lower one-third of thigh, upper and lower one-third of leg) and left lower limb (lower one-third of thigh and upper and lower one-third of leg). A significant difference (*P* < .05) was also observed in skin blood flow in digits 1 and 4 of right foot and digits 2, 4 and 5 of left foot. ANOVA results were significant (*P* < .05) for right and left foot oxygen saturation but not for heart rate and temperature. At 6 months and 1 year, the groups differed in differential segmental arterial pressure in upper third of left and right legs. Connective tissue massage improves blood circulation in the lower limbs of type 2 diabetic patients at stage I or II-a and may be useful to slow the progression of PAD.

## 1. Introduction

Peripheral arterial disease (PAD) is a common disease worldwide and is associated with a high rate of disability [1, 2]. Diabetes is one of the main causes of PAD. The development of vascular complications in diabetics depends on the length of time with the disease and their glycemia control [3]. At endothelial level, diabetic vascular complications can lead to luminal changes, affecting fibrinolysis, plasma coagulation, platelet function, and to parietal changes in contractile and secretory functions [4, 5]. Diabetics also have a diminished endothelium-dependent arterial relaxation capacity, due to self-generating changes in the generation, release, and association of self-produced vasodilatory substances [6]. Nitric oxide-mediated vasodilation is also affected, since nitric oxide is deactivated by free radicals and advanced glycation end products [7, 8].

Intermittent claudication (IC) is a transient ischemia caused by the inability of the vascular system to meet muscle metabolic requirements. It usually clinically manifests as a constrictive pain in the lower leg, although this pain can also be reported in the thigh or foot in pre-claudication conditions [9]. The pain can appear earlier during steep or fast walks or at low temperatures. Other symptoms associated with IC are cold feet and mottled hairless skin with dryness or ulcerations. IC symptoms can remain stable, heal spontaneously, or develop into chronic ischemia of the lower limbs. It is well documented that around 5% of men and 2.5% of women aged >60 years have IC symptoms [1]. Leriche-Fontaine established a four-stage clinical classification of chronic lower limb ischemia. Stage I is characterized by atheroma plaque symptoms, but blood vessel obstruction is incomplete and compensatory mechanisms have developed. In stage II, pain in lower limb muscle groups, mainly calf muscles, is triggered by walking and eases after rest: symptoms appear after a distance of >150 m in stage II-a and after <150 m in stage II-b. In stage III, the patient experiences pain while resting that worsens when the limb is raised; the pain is mainly localized in the feet, which become sensitive and cold and take on a pale or red (erythematous) appearance. In stage IV, the patient has ulcerations and limb necrosis and finds walking difficult [10]. In stages III and IV, chronic lower limb ischemia is mainly treated surgically by thrombectomy, embolectomy, fasciotomy, thromboendarectomy, bypass or amputation along with pre- and post-surgical drug treatments [11]. In stage II, treatment largely consists of diabetic control [12], non-pharmacological methods (physiotherapy, Yoga programs, antioxidant plants) [13–18], genetic therapy, and/or medication [19–21]. Patients with stage I disease receive no therapy because they show no symptoms. It is therefore important to implement measures to detect initial stages of PAD and to control risk factors [22]. A review of the literature on subclinical PAD diagnosis concluded that individuals with risk factors (obesity, cholesterol, sedentary lifestyle, smoking, dyslipemia) and positive family history should undergo vascular Doppler examination (to calculate ankle/brachial index (ABI)) and arterial plethysmographic examinations (arterial neumoplethysmography and photoplethysmography) [23–27].

Connective tissue massage (CTM) may reduce symptoms and improve IC by increasing blood circulation to the musculature. This is achieved by massaging along reflex lines on areas of the skin connected (at a distance) with deep tissue and internal organs, known as Head zones [28, 29]. The hypertonic muscle region is known as the McKenzie zone, while tissues linked to internal organs via nerve connections are called Dicke's connective zones. Stimulation of these areas generates a neuro-vegetal balance that produces a relaxed and analgesic state in the patient and a regulation of vasomotricity in areas distantly linked via nerve connections. CTM always begins in areas away from hyperalgic points in affected methameric muscles to avoid painful reactions. Loose connective tissue is stretched and stimulated by the massage to produce a neurovegetal balance, which skin-muscle reflex massage mainly achieves via the autonomous or vegetal nervous system. The stimulus produced at subcutaneous connective tissue level relaxes contracted tissues and improves the circulation due to vasodilation mediated by the vegetal nervous system [30, 31]. To our best knowledge, however, no specific evaluation has been published on the effects of CTM in patients with PAD. With this background, the objective of this study was to determine the efficacy of a CTM program to improve blood circulation and IC symptoms in the lower limb and to serve as a preventive measure against the progression of PAD in type 2 diabetic patients. This approach may be of special interest for patients unable to take part in physical exercise programs.

## 2. Methods

### 2.1. Setting and Participants

This randomized, placebo-controlled trial initially recruited all accessible patients with medical records on the computer database of a Healthcare District in Southern Spain who were diagnosed with and undergoing treatment for type 2 diabetes: a total of 146 patients. Study enrolment was from September 1 to December 20, 2005. The 146 patients underwent an initial examination (see below), and their consent was obtained to review their clinical records to gather clinical data, including medication history and date of type 2 diabetes mellitus diagnosis, defined by oral glucose tolerance test of >200 mg/dL or fasting blood glucose test of >126 mg/dL on two separate occasions during the previous year.

The physical examination was conducted after >3 h without food intake and >4 h without medication [32], measuring the ABI with the patient in supine position after a Stradness test (10% gradient treadmill at 3 km h^−1^ up to maximum tolerated walking distance) [33]. An 8 MHz Doppler probe (Hadeco Smartdop SD-20) and mercury sphygmomanometer were used for this measurement, determining systolic blood pressure (SBP) first in the right and left brachial arteries and then in the right and left posterior tibial arteries. The ABI was obtained by dividing the SBP of the posterior tibial artery in each extremity by the SBP of the brachial artery [25, 34]. The examination also yielded glycosylated hemoglobin and BMI values, calculated as weight (kg) divided by height squared (m^2^).

Study inclusion criteria were: diagnosis of type 2 diabetes; ABI of 0.6–0.9 on post-procedural Strandness test (declared equivalent to stages I and II-a of Leriche-Fontaine classification by the American Diabetes Association and American College of Cardiology) [35]; glycosylated hemoglobin of 7–10%; age of 18–65 years, and BMI of 27–35. Exclusion criteria were: PAD stage II-b or higher; peripheral venous insufficiency; cardiac, renal, or hepatic insufficiency; uncontrolled hypertension (SBP > 165/95 mmHg); central or peripheral nervous system disorders; or myopathic or neurologic damage that impaired mobility. These criteria were satisfied by 98 patients (58 females and 40 males), who gave their written consent to participation after being fully informed about the study and told that they would be able to choose the days and times of measurement and treatment sessions. The study was approved by the ethics committees of our university (University of Almeria) and hospital (Andalusian Health Service). Patients were assigned to one of two groups by using a randomized balanced (stratified) selection process. The groups were balanced for type of medication received and Leriche-Fontaine stage (I or II-a), using a stratification system that generates a sequence of letters (from table of correlatively ordered permutations) for each category and combination of categories. The assessor, massage and magnotherapy therapists, and patients were not blinded to the treatment allocation of patients. The trial was conducted between January 15, 2005 and March 30, 2008. The participants were asked to make no significant changes in diet, therapy, or daily activities during the course of the study [36].

### 2.2. Measurements

The data gathered for the selection process were considered the baseline measurements. The maximum interval between baseline data collection and start of the program was one day. The same data were collected at 30 min, 6 months, and one year after the program. At these evaluation sessions, we always took the following measurements in the order given below:

#### 2.2.1. Walking Impairment

This was assessed by administering a validated Walking Impairment Questionnaire for PAD patients that contains items on difficulties to walk a given distance and at a given speed during the previous month and on symptoms associated with walking impairment [32]. The degree of difficulty was scored as none (3 points), some (2 points), great (1 point) or as no walking activity (0 point). The walking distance score was the sum of the following points: 20 points for walking indoors, 50 points for walking 50′, 150 for 150′, 300 for 300′, 600 for 600′, 900 for 900′, and 1500 points for walking 1500′. The walking speed score was the sum of the following points: 1.5 for walking slowly, 2 for walking at average speed, 3 for walking quickly, and 5 for running or jogging. Each distance walked is used as a weighting factor in the estimation of the walking difficulty. Walking distance scores range from 0 to 34.5 and walking speed scores from 0 to 6.60 [32].

#### 2.2.2. Differential Segmental Arterial Blood Pressure

**Figure 1:**
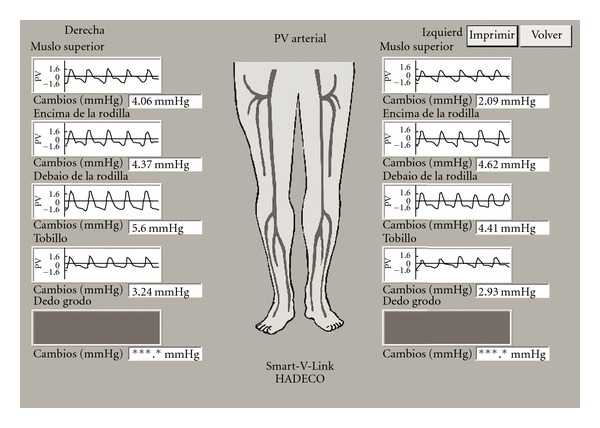
Differential segmental arterial blood pressure in lower limbs is the difference between systolic and diastolic arterial pressures in the segment under study. The wider the difference, the greater is the arterial blood flow.

This was estimated in both lower limbs using a plethysmograph (MOD-PGV-20, Smart-V-Link, Quermed, Madrid) at the proximal and distal thirds of thigh and leg; values were expressed in mmHg. Briefly, a blood pressure cuff was placed on the corresponding lower limb segment, and a pressure transducer in the plethysmograph was used to record BP changes (for 60 s) after occlusion of the venous system at 75 mmHg. The differential segmental arterial pressure is the difference between systolic and diastolic arterial pressures [27].  Figure 1 shows the plethysmography results for the limb segments.

#### 2.2.3. Differential Voltage in Skin Blood Flow

**Figure 2:**
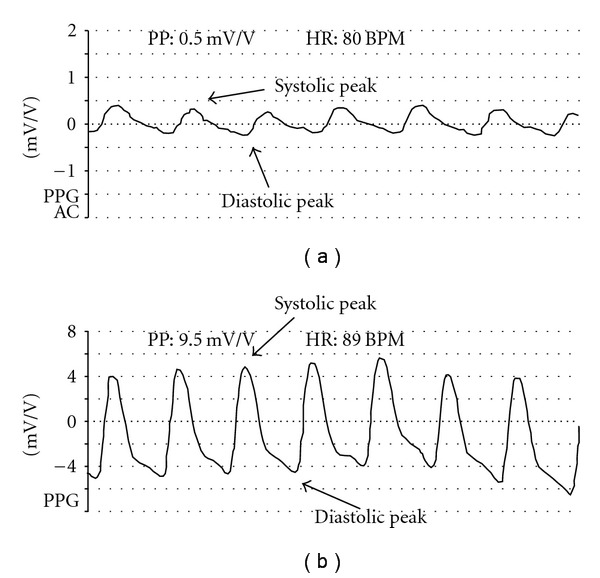
Voltage differential in skin blood flow is the difference between the voltage reached at systolic versus diastolic arterial peaks. The wider the voltage difference, the greater is the supply of arterial blood to the skin, as a consequence of vasodilation upper panel (a) shows a lower skin blood flow than that observed in lower panel (b).

Blood flow changes were measured on each digit of both feet by photo-plethysmography (MOD-PGV-20, Smart-V-Link, Quermed, Madrid), with mV/V as the unit of measurement. Photoplethysmography is based on the principle that the infrared range of light-emitting energy reflects the blood flow in subcutaneous arteries. The source of infrared transmission is the diode, which is placed next to the cadmium-selenium photosensor receiver at a pressure of 5–40 mmHg. Most of the light emitted by the diode is absorbed by the tissues, and only 5–10% reaches the subcutaneous blood vessels. The magnitude of light reflected by these blood vessels increases with higher red blood cell density [37]. The reflected energy is amplified and converted into a voltage differential. The apparatus was calibrated such that a 1% increase in blood flow corresponded to two squares on the plethysmograph thermographic paper [38–41] (Figure 2).

#### 2.2.4. Heart Rate and Oxygen Saturation

These measurements were taken with a pulseoxymeter (Megos 3300 Oxi-pulso) placed on the second digit of right and left foot after patients had rested (in supine) for 5 min. In connective tissue reflex massage, changes in heart rate (bpm) and oxygen saturation (%) are produced by the vasodilation produced.

#### 2.2.5. Skin Temperature

Skin temperature (in °C) was measured for 5 min in the inguinal region and right and left popliteal spaces using a thermographic scan (Emergen dermatempt).

### 2.3. Intervention

The massage group received a 1-h session of CTM twice a week for 15 weeks. The placebo group received a 30-min session of sham magnetotherapy in the lower back and popliteal regions (15 min per zone) twice a week for 15 weeks using disconnected equipment; these patients were instructed on the use of the magnetotherapy equipment and were unaware that it was switched off. Because all patients were treated in prone position on a massage bed with a face hole, placebo patients were unable to see the equipment during sham treatment. The therapy room was maintained at a temperature of 29.8–34.5°C (Oregon scientific model 299N) and relative humidity of 39–42% (Oregon scientific model 299N).

#### 2.3.1. Massage

The therapists applied standard therapeutic CTM, using the Dicke approach 42. The massage protocol consisted of reflex-massaging the skin with the third and fourth fingertips to stretch the subcutaneous connective tissue to the maximum. The massage must not cause pain or enter deep into structures under the connective tissue, avoiding overstimulation. The therapist flexes the elbow away from the body, rotates the shoulder internally and applies a light radial twist to the wrist. The patient should experience the massage as a “switching-off" feeling.

Before the massage, patients remained in a relaxed prone position for 30 min. At each therapy session, the full massage protocol was applied as follows. After an initial “base build-up" with lumbosacral and pelvic massage strokes [42, 43], alternate strokes from left to right were applied to the spinal axis, always in the following order [44]: five short curving movements from fifth to first lumbar vertebra, five movements at angle of lumbosacral joint, five short curving movements from intercostal proximal third to dorsal axis and up to spinal apophysis, seven light strokes in intercostal spaces, seven short curving movements from intercostal proximal third to dorsal axis and up to spinal apophysis (D_VI_ to D_VII_), five transverse intercostal movements from spine to inner edge of scapula, one deep upward movement along inner edge of scapula, one light stroke along axillary edge of scapula and a final movement along anterior side of scapular spine. The following sequence of massage strokes was then applied to the lower limbs: three strokes at origin and insertion of ischiotibial muscles, three short strokes on both sides of tensor fascia latae muscle, one lateral stroke from distal region of iliotibial band to lateral side of knee, three long strokes in triceps surae area towards each lateral side of popliteal space, three long strokes on foot from lateral edge of the calf muscles to lower edge of malleolus, three short strokes upwards from lateral edges of vastus externus and internus towards muscle fibers, two upward lateral strokes on lateral outer side of vastus externus and lateral inner side of vastus internus, four short strokes towards patella, three transversal strokes from right to the left side under tibial crest, one downward stroke from outer side of knee to fibular malleolus, five short strokes in tibioastragalar area, five short strokes on lateral outer edge of foot towards the plantar cushion, four short strokes on interosseous muscles and, finally, one long stroke from calcaneus to metatarsus-phalanx joint in plantar area. After the massage, the patient remained in a relaxed supine position for 30 min.

### 2.4. Statistical Analysis

SPSS statistics software (version 17.0) was used for statistical analyses. The reliability and validity of the model was studied by analyzing residual independence, normality, and variance homogeneity. Residual independence was analyzed by plotting the values obtained against residues, resulting in randomly distributed points showing no specific trend and therefore verifying the residual independence assumption. Residual normality was studied by using a Q-Q graph, finding the dots to be located close to the line and therefore confirming the residual normality assumption. Variance homogeneity was tested with the Levene test, obtaining a 95% confidence level and *P*-value > .05, confirming variance equality. The normal distribution of variables was determined by using the Kolmogorov-Smirnof test, expressing continuous data as means with standard deviation (SD) in the text and tables. Changes in variables within each group were measured using the paired *t*-test for independent samples. Temporal changes in the scores were examined using a two-way repeated measures ANOVA (group (massage group, placebo group) × time (baseline, 30 min, 6 months, 1 year)). Treatment efficacy was analyzed by using a *t*-test for paired samples. Independent *t*-tests were applied to baseline scores to determine whether the random assignment to groups adequately controlled for baseline demographic differences.

Relationships between variables at baseline and post-treatment were assessed by calculating the Pearson correlation coefficients, considering a 95% (*α* = 0.05) confidence level.

## 3. Results

### 3.1. Participants

**Figure 3:**
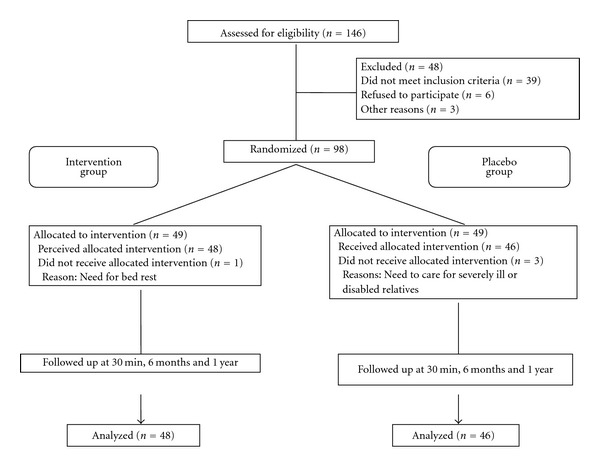
Flow chart of study participation None of the 98 randomized patients withdrew because of adverse events.

The 98 patients (58 women and 40 men) in the study group had a mean age of 53.57 ± 11.69 years (range 41–65 years). One patient dropped out of the massage group and three from the placebo group (Figure 3) due to mandatory bed rest (*n* = 1) or the need to care for severely ill relatives (*n* = 3). No patient withdrew from the study due to adverse effects of the intervention.

**Figure 4:**
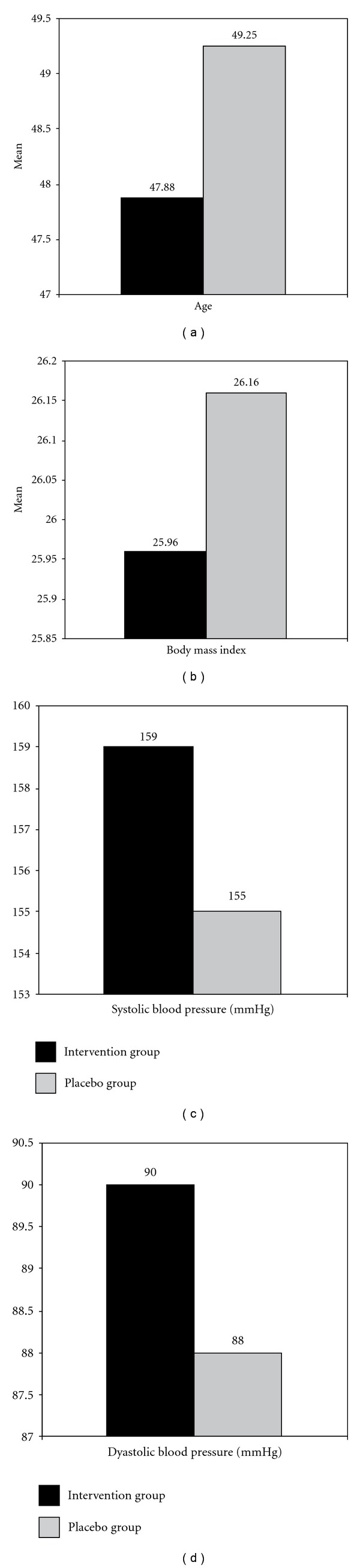
*P* < .05 between intervention and placebo groups.

No significant differences in baseline characteristics were observed between the massage and placebo groups (Figure 4).

### 3.2. 30-Min Outcomes

At 30 min after the 15-week program, mean differential segmental arterial pressure values (by plethysmography) were significantly (*P* < .05) improved versus baseline values (right and left leg) in the massage group, with the greatest improvements in upper third of the right thigh (1.69 (0.83) mmHg; *P* < .035), lower third of right thigh (2.09 (0.64) mmHg; *P* < .031), upper third of right leg (3.22 (1.15) mmHg; *P* < .029), upper third of left thigh (1.54 (0.47) mmHg; *P* < .004), lower third of left thigh (1.62 (0.94) mmHg; *P* < .021), and upper third of left leg (2.57 (1.08) mmHg; *P* < .024). No significant pressure changes were observed in the placebo group. Significant differences were found between massage and placebo group in differential arterial pressure values in the lower third of right thigh, upper third of right leg, lower third of right leg, lower third of left thigh, and upper third of left leg (Table 1). Repeated-measures ANOVA showed a significant time × groups interaction for differential arterial pressure values in lower third of right third (*F* = 8.289; *P* < .03), upper third of right leg (*F* = 7.321; *P* < .03), lower third of right leg (*F* = 11.201; *P* < .04), lower third of left thigh (*F* = 7.323; *P* < .05), and upper third of left leg (*F* = 9.321; *P* < .01).

A similar pattern was observed for the differential voltage in digits. Significant (*P* < .05) improvement from baseline in the massage group was observed in differential voltage in right first digit (3.10 (3.37) mV/V; *P* < .014), right second digit (3.93 (2.77) mV/V; *P* < .024), right fourth digit (6.50 (2.82) mV/V; *P* < .017), right fifth digit (6.30 (3.77) mV/V; *P* < .014), left second digit (6.27 (2.54) mV/V; *P* < .022), left fourth digit (5.36 (4.81) mV/V; *P* < .003) and left fifth digit (6.58 (6.13) mV/V; *P* < .001). In the placebo group, no significant change in differential voltage was observed in any digit (Table 2). ANOVA showed a significant time × groups interaction for differential voltage in right first digit (*F* = 7.984; *P* < .03), right fourth digit (*F* = 8.321; *P* < .01), left second digit (*F* = 7.224; *P* < .04), left fourth digit (*F* = 6.932; *P* < .05), and left fifth digit (*F* = 8.846; *P* < .01).

**Figure 5:**
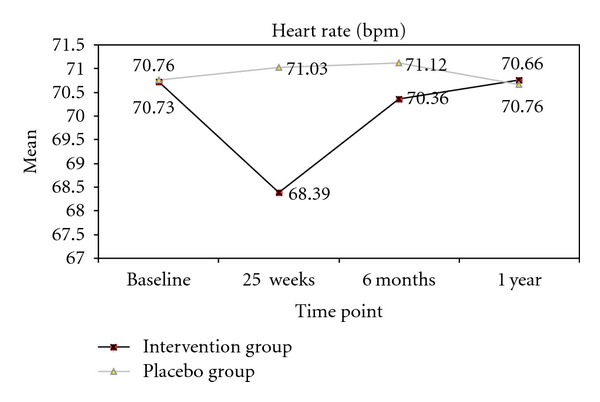
Comparisons between study groups in levels of depression, anxiety and pain. *P < .05 (95% confidence interval). Values are presented as means.

**Figure 6:**
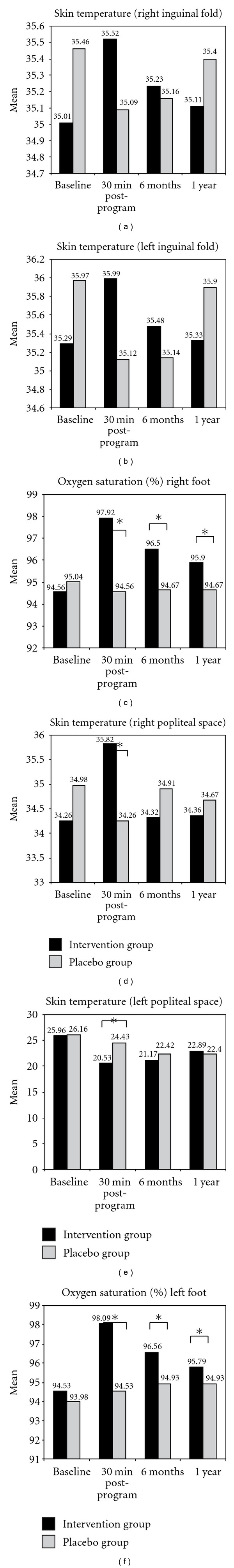
Comparisons between study groups in skin temperature and oxygen saturation. *P < .05 (95% confidence interval). Values are presented as means.

No significant changes in heart rate values were observed in either group between baseline and post-treatment (Figure 5). In the massage group, skin temperature significantly (*P* < .05) differed between baseline and the end of the 15-week treatment in right inguinal fold (35.52 (0.78)°C; *P* < .037), left inguinal fold (35.99 (0.47)°C; *P* < .048), right popliteal space (35.82 (0.39)°C; *P* < .039), and left popliteal space (35.13 (0.75)°C; *P* < .042). Significant differences were also found (baseline versus 15 wks) in oxygen saturation values in right foot (97.92 (2.93)°C; *P* < .026) and left foot (98.09 (2.64)°C; *P* < .008). The placebo group showed no significant difference in any variable between baseline and 15 weeks. The massage group showed a significantly (*P* < .05) greater improvement versus the placebo group in temperature at right and left popliteal spaces (Figure 6). ANOVA showed a significant time × groups interaction for skin temperature at right popliteal space (*F* = 5.825; *P* < .04) and left popliteal space (*F* = 4.139; *P* < .05) and for oxygen saturation in right foot (*F* = 20.034; *P* < .03) and left foot (*F* = 21.938; *P* < .01).

**Figure 7:**
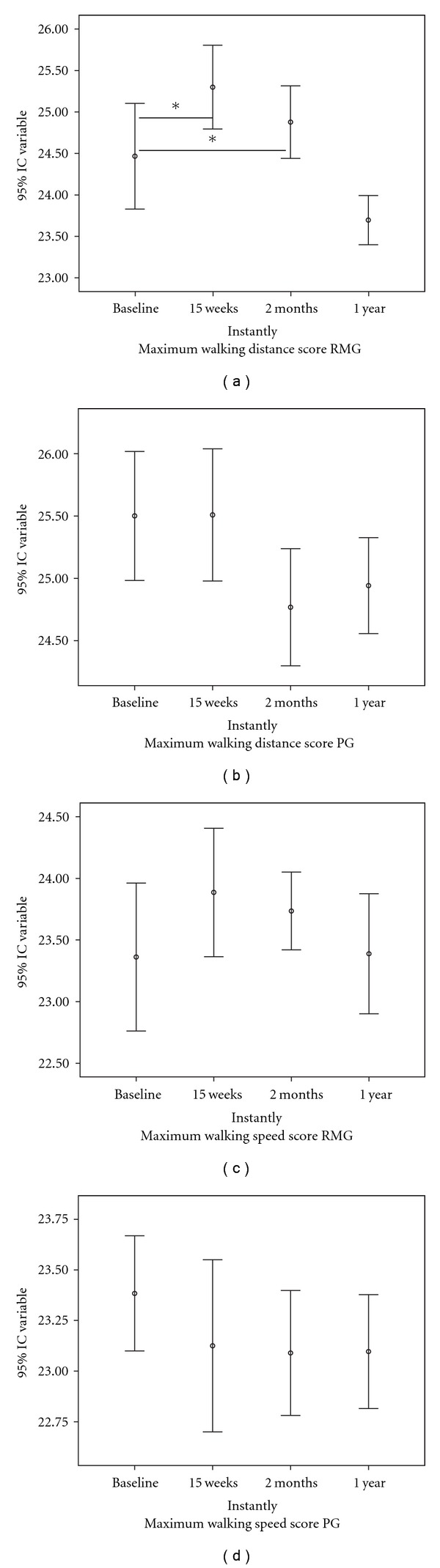
Error bars in Walking Impairment Questionnaire. RMG, reflex massage groups, PG, placebo group. *P < .05.

The mean (SD) maximum walking distance score significantly improved between baseline and 10-min post-treatment (20.03 (4.32) versus 26.85 (4.26); *P* < .029) in the massage group. There were also significant differences between groups in this score at the end of the 15-week treatment (baseline: 20.03 (4.32) versus 21.19 (3.94), *P* < .063; 15 weeks: 26.85 (4.26) versus 21.77 (3.67), *P* < .034). However, no significant change was observed versus baseline or between groups in relation to the maximum walking speed score (baseline: 3.97 (1.10) versus 3.89 (1.25), *P* < .319; 15 weeks: 4.46 (0.97) versus 3.99 (1.08), *P* < .087) (Figure 7). ANOVA showed differences in maximum walking distance score (*F* = 19.347; *P* < .03).

Although both differential segmental arterial pressure and heart rate responded significantly to CTM therapy, there were no statistically significant correlations (Pearson correlation coefficient) between the increase in skin temperature values and the decrease in heart rate values in the massage group (*r* = 0.334; *P* = .188) or the placebo group (*r* = 0.319; *P* = .721). However, there were significant correlations in the massage group between the increase in skin temperature and oxygen saturation values (*r* = 0.346, *P* = .045) and between the increase in skin temperature and differential segmental arterial pressure values in the upper third of the thigh (*r* = 0.496; *P* = .031), lower third of thigh (*r* = 0.511; *P* = .027), upper third of leg (*r* = 0.436; *P* = .025), and lower third of leg (*r* = 0.392; *P* = .046).

### 3.3. Six-Month Outcomes

At 6 months post-treatment, differential arterial pressure remained high in the CTM in segments with larger muscular mass, that is, lower third of the right thigh (*P* < .048), upper third of right leg (*P* < .045), and upper third of left leg (*P* < .039). No significant change (versus baseline) was observed in the placebo group. At 6 months, the groups significantly (*P* < .05) differed in differential pressure in lower third of right thigh, upper third of right leg, lower third of left thigh, and upper third of left leg (Table 1). There were also statistically significant (*P* < .05) differences between the groups in differential voltage in right first digit, right fourth digit, left second digit, left fourth digit, and left fifth digit (Table 2). Repeated-measures ANOVA showed a significant time × groups interaction for differential arterial pressure values in lower third of right third (*F* = 7.321; *P* < .04), upper third of right leg (*F* = 7.146; *P* < .04), lower third of left thigh (*F* = 6.852; *P* < .05), and upper third of left leg (*F* = 7.049; *P* < .04) and for differential voltage in right first digit (*F* = 8.129; *P* < .04), right fourth digit (*F* = 7.646; *P* < .03), left second digit (*F* = 8.731; *P* < .04), left fourth digit (*F* = 8.427; *P* < .04), and left fifth digit (*F* = 8.633; *P* < .04).

In the massage group, significant differences were found between baseline and six months post-treatment in skin temperature of right inguinal fold (*P* < .046) and in oxygen saturation of right (*P* < .045) and left foot (.043). The placebo group showed no significant difference between baseline and two months in any variable. The massage group showed a significantly greater improvement versus placebo group in maximum walking distance (*P* < .049) but not in maximum walking speed (*P* < .073) (Figure 7). ANOVA analysis showed significant differences for oxygen saturation in right foot (*F* = 3.821; *P* < .04) and left foot (*F* = 4.053; *P* < .03) and for maximum walking distance (*F* = 17.721; *P* < .04).

### 3.4. One-Year Outcomes

At the 1-year follow-up, the improvements observed in the massage group at 6 months largely persisted, with significant differences versus baseline in: differential pressure in upper third of right leg (*P* < .041) and upper third of left leg (*P* < .039); differential voltage in right first digit (*P* < .045) and left second digit (*P* < .046); and oxygen saturation in right (*P* < .047) and left (*P* < .049) feet. The placebo group again showed no differences with baseline values. The massage group showed a significantly greater improvement versus placebo group (*P* > .05) in differential pressure in upper third of right leg and upper third of left leg and in oxygen saturation in both feet (Table 1 and Figure 6). No significant changes were observed versus baseline or between groups in maximum walking distance or maximum speed scores (Figure 7). ANOVA analysis showed significant interaction for differential arterial pressure values in upper third of right leg (*F* = 7.124; *P* < .04) and upper third of left leg (*F* = 6.993; *P* < .04) and for oxygen saturation in right foot (*F* = 3.747; *P* < .04) and left foot (*F* = 4.236; *P* < .03).

## 4. Discussion

In a group of type 2 diabetes patients with stage I or II-a PAD, the application of a CTM protocol (Dicke approach) twice a week for 15 weeks increased differential segmental pressure in leg as measured by plethysmography, improved skin blood flow as determined by photoplethysmography, and increased pulse volume according to Doppler studies. The improvement observed in the massage group after CTM was in line with the results obtained by Castro et al. [43] applying a massage protocol to healthy subjects. Massage of the connective tissue reduces peripheral vascular resistance at microcirculation level [28–30]. The vascular structure facilitates the work of increasing the blood flow from terminal arterioles until it progressively reaches the large blood vessels. A contribution to this ascending vasodilatation may be made by the endothelium from flattened squamous cells on the contact surface between vessel wall and circulating blood. These cells respond to changes in humoral conditions in the cardiovascular system, translating these changes into vasoactive signals that regulate the blood flow [44, 45].

We measured variations in skin blood flow by photoplethysmography based on techniques studied by Didier et al. [46], Allen et al. [27] and Bertino et al. [47], who all stressed the usefulness of digital photoplethysmographic evaluation at the different Leriche-Fontaine stages of the disease. This method allows qualitative variations in wave morphology (differences between systolic and diastolic peaks) to be measured before and after therapy. The presence of arterial ischemia produces qualitative changes in curve morphology recorded by the photoplethysmograph as a differential voltage decrease versus the contralateral foot.

At the first post-therapeutic evaluation (at 30 min), a slight temperature increase due to skin vasodilation was detected, likely due to a combination of both neuronal and localized processes [48]. Previous studies [49–51] found no difference between high- and normal-blood pressure groups in the internal body temperature at which vasodilation begins.

Oxygen saturation (by pulseoxymetry) and digit ABI are good screening tools for diagnosing and monitoring PAD in type 2 diabetic patients [52]. One study found no significant improvements in oxygen saturation values in these patients after 5 min of foot massage, which may be too brief to achieve benefits; the massage produced a downward trend in the heart rate due to vasodilation [53]. In the present study, heart rate values were significantly lower versus baseline in the massage group but not in the placebo group.

It has been reported in general terms that CTM may cause patients some discomfort [54]. None of our patients described any such feelings during our application of the Dicke method. Patients were instructed to tell the therapist immediately pain was felt, and this never proved necessary. Nevertheless, discomfort may have gone unreported by some patients, and it would be useful to explore this aspect in greater depth in future studies.

### 4.1. Study Limitations

Among study limitations, the patients with type II-a PAD continued to receive their usual medication during the massage program, for ethical reasons. A further limitation is that the therapists were not blinded to the group to which the patients belonged, which may have influenced outcomes.

## 5. Conclusion

CTM increases blood circulation in the lower limbs of type 2 diabetic patients at stage I and II-a (Leriche-Fontaine) of the disease, with improvements in differential segmental pressure in leg and greater skin blood flow. At 30 min after this massage, the heart rate lowered and oxygen saturation and temperature values rose, confirming the role of the parasympathetic nervous system in the effects of this treatment. CTM is a treatment option for asymptomatic patients suspected of being in Leriche-Fontaine stage I, that is, having the main risk factors for developing the disease. This type of massage may also be useful to improve symptoms and perhaps slow the progression of the disease in stage II-a PAD patients who have difficulties in taking part in any kind of exercise, including walking programs.

## Figures and Tables

**Table 1:** Differences between groups in differential segmental arterial pressure in lower limbs.

Variable (mmHg)	Baseline *M* (SD)		*P* value Pre-T	30 min post-program *M* (SD)		*P* value 1st-PT	6 months *M* (SD)		*P* value 2nd-PT	1 year *M* (SD)		*P* value 3rd-PT
	IG	PG		IG	PG		IG	PG		IG	PG	
UTRT	1.09 (0.44)	1.17 (0.32)	.32	1.69 (0.83)	1.21 (0.76)	.07	1.50 (0.49)	1.24 (0.29)	.09	1.48 (0.33)	1.19 (0.41)	.11
LTRT	1.49 (0.06)	1.22 (0.12)	.10	2.09 (0.64)	1.15 (0.51)	.03*	1.87 (0.55)	0.89 (0.83)	.04*	1.56 (0.94)	1.06 (0.36)	.07
UTRL	2.31 (0.97)	1.79 (0.72)	.07	3.22 (1.15)	1.88 (0.67)	.03*	2.91 (1.05)	1.87 (0.58)	.04*	2.49 (0.61)	1.86 (0.90)	.04*
LTRL	0.90 (0.06)	0.60 (0.97)	.99	1.21 (0.11)	0.57 (0.06)	.05*	1.18 (0.99)	0.69 (0.64)	.08	1.03 (0.43)	0.64 (0.96)	.09
UTLT	1.05 (0.34)	1.12 (0.82)	.35	1.54 (0.47)	1.09 (0.71)	.08	1.31 (0.58)	1.11 (0.45)	.15	1.26 (0.23)	1.08 (0.55)	.23
LTLT	1.39 (0.73)	1.13 (0.79)	.10	1.62 (0.94)	0.99 (1.01)	.05*	1.54 (0.88)	0.86 (0.72)	.05*	1.41 (0.62)	0.98 (0.34)	.08
UTLL	1.66 (2.57)	1.32 (0.99)	.98	2.57 (1.08)	1.33 (0.92)	.01*	2.21 (0.83)	1.35 (0.64)	.04*	1.98 (0.51)	1.29 (0.63)	.04*
LTLL	0.89 (0.31)	0.92 (0.87)	.67	1.12 (0.57)	0.97 (0.56)	.18	1.07 (0.46)	0.94 (0.38)	.25	0.99 (0.712)	0.90 (0.57)	.36

Values are presented as mean (SD). RMG, reflex massage group; PG, placebo group; 1st PT, post-treatment (30 min after 15-week treatment ends); 2nd PT, 6 months post-treatment; 3rd PT, 1 year post-treatment; UTRT, upper third of right thigh; LTRT, lower third of right thigh; UTRL, upper third of right leg; LTRL, lower third of right leg; UTLT, upper third of left thigh; LTLT, lower third of left thigh; UTLL, upper third of left leg; LTLL, lower third of left leg.

*P < .05 was considered significant.

**Table 2:** Differences between groups in differential voltage in skin blood flow.

Variable (Digits) (mV/V)	Baseline *M* (SD)		*P* value Pre-T	30 min post-program *M* (SD)		*P* value 1^a^-PT	6 months *M* (SD)		*P* value 2^a^-PT	1 year *M* (SD)		*P* value 3^a^-PT
	IG	PG		IG	PG		IG	PG		IG	PG	
Right first	2.19 (2.60)	2.23 (4.23)	.35	3.10 (3.37)	2.18 (3.89)	.03*	3.04 (6.55)	2.29 (5.60)	.04*	2.59 (4.13)	2.24 (4.97)	.06
Right second	3.10 (1.94)	3.42 (2.35)	.07	3.93 (2.77)	3.56 (2.27)	.06	3.41 (4.73)	3.48 (2.11)	.30	3.21 (5.03)	3.33 (3.01)	.27
Right third	2.63 (0.12)	2.87 (1.45)	.10	2.96 (3.19)	2.76 (2.03)	.14	2.24 (5.24)	2.80 (1.63)	.05	2.38 (6.04)	2.71 (1.98)	.08
Right fourth	4.73 (1.08)	4.67 (2.13)	.29	6.50 (2.82)	4.62 (1.99)	.01*	5.73 (4.46)	4.58 (4.32)	.03*	5.02 (3.12)	4.52 (3.12)	.05
Right fifth	5.57 (2.69)	6.01 (2.56)	.06	6.30 (3.77)	6.15 (3.11)	.08	6.59 (4.80)	6.23 (2.88)	.06	6.01 (5.32)	6.08 (2.97)	.29
Left first	3.84 (0.37)	4.12 (1.35)	.07	4.17 (2.53)	4.09 (1.41)	.31	4.55 (4.32)	4.21 (1.57)	.08	4.11 (5.32)	4.01 (1.22)	.22
Left second	5.20 (1.50)	5.39 (3.78)	.16	6.27 (2.54)	5.55 (3.88)	.04*	6.12 (4.17)	5.45 (3.92)	.04*	5.88 (4.01)	5.39 (3.61)	.05
Left third	4.06 (2.89)	4.23 (2.57)	.21	4.44 (2.31)	4.17 (2.78)	.09	4.52 (2.95)	4.29 (2.65)	.09	4.48 (1.12)	4.25 (2.82)	.10
Left fourth	4.88 (4.03)	4.92 (4.99)	.35	5.36 (4.81)	4.89 (4.83)	.05*	5.44 (3.73)	4.98 (5.32)	.04*	5.24 (2.39)	4.96 (5.05)	.09
Left fifth	4.72 (5.01)	4.51 (4.57)	.12	6.58 (6.13)	4.49 (4.68)	.01*	5.04 (4.13)	4.57 (4.08)	.04*	4.69 (3.13)	4.52 (4.14)	.13

Values are presented as mean (SD). RMG, reflex massage group; PG, placebo group; 1^a^ PT, post-treatment (30 min after 15-week treatment ends); 2^a^ PT, 6 months post-treatment; 3^a^ PT, 1 year post-treatment).

*P < .05 was considered significant.
